# Accuracy of Patient Height, Weight and Ideal Body Weight Estimates in the Emergency Department

**DOI:** 10.51894/001c.5934

**Published:** 2017-02-02

**Authors:** Kevin Boehm, Cassie Welt, Jeanette Grimaldi

**Affiliations:** 1 St. Mary Mercy, Dept. of Emergency Medicine and Graduate Medical Education, Livonia, MI; Michigan State University, College of Osteopathic Medicine, East Lansing, MI; 2 Henry Ford Wyandotte Hospital, Dept. of Emergency Medicine, Wyandotte MI; Mercy Medical Center, Clinton, IA; 3 Henry Ford Wyandotte Hospital, Dept. of Emergency Medicine, Wyandotte MI; Beaumont Wayne, Wayne, MI

**Keywords:** clinical estimates, ideal body weight, estimated body weight

## Abstract

**CONTEXT:**

The purpose of this study was to establish the accuracy of emergency medicine professionals’ estimates of a sample of patients’ heights, weights and ideal body weights (IBW).

**METHODS:**

This was a cross-sectional survey study with 69 emergency medicine professionals concerning estimates of five standardized patients. Board-certified emergency physicians, emergency medicine residents and emergency department nurses were asked to estimate patient height, weight and (IBW) by looking at a series of photographs of five standardized patients with varied body types. Repeated measure analysis of variance procedures were used to examine for estimates-to-actual measurement differences.

**RESULTS:**

Overall height, weight and IBW estimate differences did vary significantly for the majority of the five standardized patients. Respondents’ clinical position (i.e., attending physician, resident, or nurse) and years of clinical experience did show a significant level of influence on estimate-to-actual differences for some proportion of patient estimates.

**CONCLUSION:**

Our results support the common belief that the accuracy of overall weight and height estimates in emergency department settings is unacceptably low, and that a patient’s stated weight and height is likely to be more accurate. Further work is required in this area of emergency medicine practice since these types of inaccuracies could potentially compromise the effectiveness of therapies and treatments during emergent situations.

## INTRODUCTION

The number of annual emergency department (ED) visits in the US is on the rise, reaching over 127 million visits in 2010.^1^ Patients frequently present in the ED with emergent scenarios requiring rapid administration of lifesaving medications before there is time for them to be accurately weighed. Critically ill patients may also present with an altered mental status or inability to bear weight due to their injuries. Since many medications used in such emergent settings are dosed by either weight or *ideal body weight* (IBW), inaccurate weight estimates by ED professionals can lead to inappropriate medical therapies.^2^ Key medications requiring this type of information include heparin, thrombolytics, phenytoin, and rapid sequence inductions agents such as rocuronium and ketamine.^2^

### Purpose

The purpose of this cross-sectional study was to examine the accuracy of a sample of emergency medicine professionals’ estimates of a series of standardized patients’ height, weight and IBW compared to actual measurement readings.

## METHODS

### Study Setting

The ED of Henry Ford Wyandotte Hospital is an academic community-based hospital with over 70,000 annual visits. During a two-week period in 2014, a sample of board-certified emergency medicine attending physicians, residents and registered nurses practicing at the time of the study were surveyed.

### Study Design

Before data collection, the study protocol was reviewed and approved by the Henry Ford Health System international review board. The authors administered a cross-sectional survey that they had created to ED staff at a community-based academic hospital as well as an affiliated standalone ED in the community. To generate photographs of the five standardized patients, five healthy adult volunteers with varied body types were recruited. After obtaining consent from each subject, a survey of 10 photographs of the five *standardized* patients was constructed and distributed to a convenience sample of all ED attending physicians, residents, and registered nurses. No specific inclusion or exclusion criteria were observed for enrollment of these standardized patients.

Each patient’s height and weight were documented using the same scale and measuring tape at the same time of day. Patient heights were calculated in inches and weight in pounds. Their IBW was then calculated from these data using the following formulae: Males: IBW = 50 kg. + 2.3 kg. for each inch height over 5 feet. Females: IBW = 45.5 kg. + 2.3 kg. for each inch height over 5 feet. The images of each patient were taken at two angles, lying on the same ED stretcher, and wearing the same type of patient gown.

The faces of standardized patients were obscured to maintain their anonymity. All photographic images were inserted onto a survey sheet and disseminated to ED attendings, residents and nurses during the weeks of 1/29/2014 through 2/7/2014. Participating ED professionals were asked to provide estimates of each standardized patient’s height, weight and IBW based on the photos. Participating respondents were also asked to report their position in the ED (i.e., attending, resident or nurse) and years in clinical practice. Years of practice were categorized into PGY 1-4 (if resident), and less than five years, between five and 10 years, or greater than 10 years (if attending physician or nurse).

A total of 69 surveys were collected, including 14 attending physicians, 18 ED residents, and 37 registered nurses. Survey data were entered into an electronic spreadsheet by two of the authors (CW, JG). All survey data were initially entered by one researcher and rechecked by a second researcher to ensure accuracy. The height, weight and IBW estimations were then compared to actual measured values to calculate estimate-to-actual (ETA) differences.

### Data Analysis

ETA difference data were analyzed as a series of continuous outcome variables for height, weight, and IBW. A series of repeated measure analysis of variance (ANOVA) analytic procedures were used to assess the level of influence of respondents’ position in the ED and clinical experience category on overall standardized patient individual height, weight and IBW estimates as well as ETA differences.

## RESULTS

The overall results for patient estimates and ETA values are depicted in Tables 1 through 3. The first table reports the overall and mean ETA height measurement differences. The second table depicts weight measurement patterns, and Table 3 depicts IBW data results. In each of these tables, statistically significant results are denoted by bolding of the p-values. 

**Table 1:** Differences in Height Estimates (N = 69)

	**Patient A**	**Patient B**	**Patient C**	**Patient D**	**Patient E**	**Patients A-E**
**Actual Measured Height (in.)**	**76.0**	**73.0**	**75.5**	**64.5**	**75.0**	
**Height** Difference* **Mean ± SD**						
**Overall Differences**						
Height difference	-2.2 ± 1.4	-4.5 ± 2.9	-2.3 ± 1.8	0.3 ± 2.5	-2.6 ± 2.1	-2.3 ± 1.4
p-value (t-test)**	**<0.001**	**<0.001**	**<0.001**	0.325	**<0.001**	**<0.001**
**Differences by Position**						
RN	-2.2 ± 1.4	-4.3 ± 2.8	-2.6 ± 2.0	0.0 ± 2.5	-2.3 ± 2.5	-2.3 ± 1.5
EM resident	-2.2 ± 1.5	-4.8 ± 3.1	-1.9 ± 1.8	0.2 ± 2.5	-3.1 ± 1.3	-2.4 ± 1.0
EM attending	-2.8 ± 1.8	-4.7 ± 3.2	-1.9 ± 1.1	1.1 ± 2.4	-2.4 ± 2.0	-2.2 ± 1.4
p-value (t-test)**	0.289	0.549	0.285	0.280	0.302	0.968
**Differences by Clinical Experience**						
PGY	-2.2 ± 1.5	-4.8 ± 3.1	-1.9 ± 1.8	0.2 ± 2.5	-3.1 ± 1.3	-2.4 ± 1.0
< 5 years	-2.5 ± 1.4	-4.1 ± 2.7	-2.6 ± 1.5	0.5 ± 2.6	-3.0 ± 1.8	-2.4 ± 1.4
5-10 years	-2.6 ± 1.4	-4.5 ± 3.5	-2.7 ± 1.9	0.0 ± 2.3	-3.2 ± 3.0	-2.6 ± 1.4
> 10 years	-1.9 ± 1.8	-4.8 ± 2.8	-2.1 ± 2.2	0.4 ± 2.7	-0.9 ± 1.9	-1.9 ± 1.6
p-value (t-test)**	0.591	0.901	0.573	0.968	**0.002**	0.437

*refers to difference between estimated and actual height

**statistically significant values (bolded) suggest a low probability that result observed would have occurred if no true correlation, (i.e., ρ (rho)=0)

**Table 2:** Differences in Weight Estimates (N = 69)

	**Patient A**	**Patient B**	**Patient C**	**Patient D**	**Patient E**	**Patients A-E**
**Actual Measured Weight (lbs.)**	**241.0**	**170.0**	**191.0**	**143.0**	**285.0**	
**Weight** Difference* **Mean ± SD**						
**Overall Differences**						
Weight difference	-22.2 ± 25.2	-4.0 ± 19.0	-0.9 ± 22.7	19.0 ± 24.6	-51.8 ± 31.2	-12.0 ± 15.3
p-value (t-test)**	**<0.001**	0.083	0.744	**<0.001**	**<0.001**	**<0.001**
**Differences by Position**						
RN	-15.3 ± 23.5	-0.8 ± 19.9	-1.6 ± 24.3	21.8 ± 28.4	-43.6 ± 33.8	-7.9 ± 15.0
EM resident	-26.6 ± 28.0	-7.5 ± 15.4	-1.9 ± 20.2	12.8 ± 18.3	-61.1 ± 30.2	-16.9 ± 15.2
EM attending	-34.9 ± 20.4	-7.9 ± 20.3	2.2 ± 22.8	19.8 ± 20.2	-61.4 ± 18.1	-16.5 ± 13.9
p-value**	**0.010**	0.105	0.940	0.893	**0.013**	**0.024**
**Differences by Clinical Experience:**						
PGY	-26.6 ± 28.0	-7.5 ± 15.4	-1.9 ± 20.2	12.8 ± 18.3	-61.1 ± 30.2	-16.9 ± 15.2
< 5 years	-24.5 ± 23.8	-2.4 ± 24.8	-1.6 ± 26.3	23.5 ± 28.2	-52.5 ± 22.8	-11.5 ± 15.0
5-10 years	-22.7 ± 23.5	4.6 ± 15.4	2.9 ± 27.1	20.8 ± 22.1	-38.3 ± 37.6	-6.6 ± 16.7
> 10 years	-14.3 ± 25.2	-8.5 ± 14.4	-1.5 ± 18.3	18.6 ± 27.5	-50.6 ± 35.8	-11.3 ± 14.4
p-value**	0.247	0.632	0.729	0.844	0.190	0.241

*refers to difference between estimated and actual weight

**statistically significant values (bolded) suggest a low probability that result observed would have occurred if no true correlation, (i.e., ρ (rho)=0)

**Table 3:** Differences in Ideal Body Weight (IBW) Estimates (N = 69)

	**Patient A**	**Patient B**	**Patient C**	**Patient D**	**Patient E**	**Patients A-E**
**Actual Calculated IBW (lbs.)**	**189.0**	**165.0**	**188.0**	**126.0**	**186.0**	
IBW Difference* **Mean ± SD (in/lbs)**						
**Overall:**						
IBW difference	8.4 ± 20.6	-15.9 ± 17.6	-2.2 ± 21.3	9.8 ± 15.4	6.0 ± 22.4	1.2 ± 13.5
p-value (t-test)**	**<0.001**	**<0.001**	0.391	**<0.001**	**0.031**	0.459
**By staff:**						
RN	15.1 ± 19.2	-14.0 ± 18.3	-2.5 ± 21.3	10.0 ± 15.4	14.1 ± 24.2	4.6 ± 13.4
EM resident	-3.7 ± 21.7	-20.3 ± 14.1	-5.5 ± 22.2	3.7 ± 13.0	-7.7 ± 15.9	-6.7 ± 10.8
EM attending	6.4 ± 15.7	-15.6 ± 19.7	2.6 ± 20.8	17.2 ± 16.2	1.9 ± 14.8	2.5 ± 13.6
p-value**	**0.031**	0.345	0.829	0.407	**0.009**	0.170
**By experience:**						
PGY	-3.7 ± 21.7	-20.3 ± 14.1	-5.5 ± 22.2	3.7 ± 13.0	-7.7 ± 15.9	-6.7 ± 10.8
< 5 years	13.0 ± 22.4	-14.9 ± 20.7	-0.1 ± 22.9	14.7 ± 18.5	3.5 ± 18.9	3.3 ± 15.8
5-10 years	10.2 ± 15.2	-10.0 ± 22.7	2.0 ± 24.4	8.4 ± 12.8	20.7 ± 34.2	6.3 ± 15.8
> 10 years	14.1 ± 15.8	-16.9 ± 11.8	-4.5 ± 16.4	11.1 ± 14.0	13.1 ± 12.8	3.4 ± 7.5
p-value**	**0.021**	0.329	0.492	0.204	**<0.001**	**0.008**

*refers to difference between estimated and actual measure of ideal body weight

**statistically significant values (bolded) indicate a low probability that result observed would have occurred if no true correlation, (i.e., ρ (rho)=0)

As Table 1 demonstrates for height, while there were statistically significant variations observed for overall height estimate differences in four of five patients, as well as the combined estimate from all five patients, the significance of height estimate variations was lost when controlling for position subgroups (i.e., EM attending, EM resident, and nurse), and by levels of clinical experience (i.e., PGY year, <5 years, 5-10 years, and > 10 years).

Table 2 summarizes the weight estimate results, with a statistically significant variation in ETA differences observed from three standardized patients. Similarly, statistically significant variations were observed for ETA weight estimates by position, although not by clinical experience levels. Table 3, which depicts IBW estimate patterns, demonstrates somewhat similar patterns of significance to Table 2 for overall, EM position and across clinical experience category estimate differences.

## DISCUSSION

Many of the calculations and medications routinely used in the ED require that professionals possess accurate information concerning patients’ weight and height. Many times, rushed ED professionals may feel obliged to make such estimates due to patients’ presenting circumstances. Current research in the adult population has shown that patients are historically more accurate at estimating their own weight than ED staff.^2^

Earlier studies comparing attending physicians, resident physicians, nurses, paramedics and patients estimates to actual weight measurements have suggested that ED professional estimates are frequently quite inaccurate.^3,4^ In fact, one study group concluded that patients were almost nine times more likely to accurately estimate their own actual weight than providers.^4^

Two similar studies have reported that patients were generally more accurate in estimating their true weight, with healthcare workers showing only a moderate degree of accuracy when patients’ personal weight estimates were compared to providers.^2,5^ Specifically, it was specifically reported in another project that ED staff provided accurate weight in only 33% of estimates, with such estimates even less accurate for underweight and overweight patients.^6^

The results of the authors’ study in a typical community-based ED setting were somewhat mixed. As seen in Table 1 through 3, sample professionals’ overall height, weight, and IBW estimates varied at statistically significant levels for the majority of standardized patients. Results also indicated that estimates were significantly variable amongst staff for some patients based on position in the ED and amount of clinical experience. 

Of course, there were several limitations to our study. First, our use of photographs offered only a two-dimensional image of the standardized patients.  Obscuring the patients’ faces in photographs could have limited how accurately participating providers estimated patients’ weights and/or heights. Moreover, we have subsequently concluded that many ED employees actually prefer to interact directly with patients to make more accurate estimates. Finally, the generalizability of our results to other settings may be limited due to our smaller study sample. A larger sample may have enabled us to detect more statistically significant ETA sample subgroup differences. 

## CONCLUSION

In spite of these limitations, this study demonstrated the high variability and inaccuracy of weight and height estimations typically made by ED employees, regardless of their position or years of clinical practice. Our results and conclusions support our theory that overall estimates of weight and height in ED settings are unacceptably low, and that a patient's stated weight and height may tend to be more accurate. Further work is required in this area of emergency medicine practice since these types of inaccuracies could potentially compromise effective therapies and treatments during emergent situations.

### Conflict of Interest

The authors declare no conflict of interest.
